# Nonpharmacological Interventions for the Treatment of Cardiometabolic Risk Factors in People With Schizophrenia—A Systematic Review

**DOI:** 10.3389/fpsyt.2019.00566

**Published:** 2019-08-16

**Authors:** Ewa Tumiel, Adam Wichniak, Marek Jarema, Michał Lew-Starowicz

**Affiliations:** ^1^III Department of Psychiatry, Institute of Psychiatry and Neurology, Warsaw, Poland; ^2^Department of Psychiatry, Centre of Postgraduate Medical Education, Warsaw, Poland

**Keywords:** schizophrenia, metabolic syndrome, cardiometabolic risk factors, exercise, diet, psychotherapy

## Abstract

**Background:** People suffering from schizophrenia are notably vulnerable to cardiometabolic risk factors (CMRF), such as obesity, high blood pressure, hyperglycemia and insulin resistance, high serum triglycerides, and low serum high-density lipoprotein (HDL), which are related to increased mortality and decreased quality of life. The increased risk of “metabolic syndrome” (MS) is related to low physical activity, an unhealthy diet, and side effects of antipsychotic drugs. Nonpharmacological interventions seem to be important in the prevention and therapy of MS.

**Aim:** This paper provides an overview of published studies and a critical analysis of pilot programs involving nonpharmacological measures aimed at prevention and treatment of CMRF in patients with schizophrenia.

**Material and Method:** We searched the PubMed, PsycARTICLES, and Cochrane Library databases to identify clinical trials. We included full-text studies that met the following criteria: age > 18 years, a diagnosis of schizophrenia or schizoaffective disorder, and monitored parameters associated with MS.

**Results:** All 1,555 references were evaluated for inclusion in the review, and 20 met the inclusion criteria. Nonpharmacological interventions led to improvement in physical health and showed a promising potential for implementation in treatment programs dedicated to this particular group of patients. However, a critical analysis revealed limitations, which have implications for the direction of future research.

**Conclusions:** Patients suffering from schizophrenia can benefit from nonpharmacological interventions aimed at counteracting CMRF, improving either metabolic parameters, cardiovascular fitness, or their health perception. Notwithstanding, to achieve long-term effects, future studies should comprise appropriate follow-up procedures.

## Introduction

Schizophrenia is associated with significant reduction in health-related quality of life. Meta-analyses reveal significant health differences between people suffering from schizophrenia and the general population. Patients with schizophrenia (PwS) are two to three times more likely to have a higher mortality rate and a reduced lifespan expectancy of 13–30 years. This also applies in countries where the quality of the medical care system is generally considered to be good. Sixty percent of mortality among PwS is due to complications of metabolic and cardiovascular diseases. Weight gain applies to 72% of patients taking APs, whereas 42–60% of PwS are obese. Metabolic syndrome affects 19.4–68% of patients treated for schizophrenia (differences depend on the country, gender, age, ancestry, and medication). The risk of developing type 2 diabetes, one of the components of MS, is estimated to be two to three times higher than in the general population and nearly half of patients remain untreated (1). A higher risk of cardiovascular complications is commonly related to a high frequency of diabetes, hypertension, smoking, poor nutrition, obesity, impaired lipid profile, and low physical activity (2, 3), but also with the use of antipsychotics (APs). It has been established that treatment with APs, particularly the second generation, largely contribute to metabolic complications. However, even before the introduction of new medications, Henry Maudsley noted a relationship between diabetes and schizophrenia (4). Many studies have shown that a large proportion of untreated patients are characterized by impaired glucose tolerance (and the same applies to siblings of patients with schizophrenia), as well as increased distribution of visceral fat or insulin resistance (5–7). Recent studies have indicated that diabetes and schizophrenia may share a common genetic background (8). It has also been shown that people suffering from schizophrenia are significantly less active compared to a control group (2, 3). In addition, it is important to note that metabolic risk factors are often present in patients with a first-episode psychosis (FEP) (9–11). This may be related to specific symptoms of schizophrenia: negative symptoms, depressive symptoms, and impaired cognitive function (2). Importantly, there is significant disparity in access to health-care services in this group of patients. It seems reasonable to state that the problem of comorbidities and premature mortality among PwS requires interventions by representatives of mental health care, as this is often the only medical care used by this group of patients. Clinical guidelines underline the importance of regular monitoring of metabolic and cardiovascular parameters and recommend nonpharmacological interventions, such as physical activity, diet, or psychotherapy. Recently published reviews evaluated the quality of guidelines for cardiovascular risk in people with schizophrenia. The authors acknowledged a significant variability in the guidelines and a need for further investigation (12, 13). Most of the guidelines recommended monitoring the following measurements (in order of frequency): fasting glucose, body mass index, triglycerides, total cholesterol, waist, high-/low-density lipoprotein, blood pressure, and symptoms of diabetes (12). Moreover, in terms of nonpharmacological interventions, most guidelines recommended regular physical activity, diet, psychoeducation, treatment of metabolic abnormalities, and smoking cessation (12, 13). Authors commonly claim that assessments should be repeated 6 and 12 weeks after initiation of a new AP drug treatment (12). The intention of the guidelines is to improve cooperation and shared care between different health-care professionals and to increase awareness among psychiatrists and primary care physicians about the need to screen and treat cardiovascular risk factors and diabetes (14). Research conducted both among the general population and people with mental disorders has consistently shown the impact of nonpharmacological interventions in reducing the risk of cardiovascular disease (15). People suffering from schizophrenia may require a greater effort in the prevention of obesity and related comorbidities.

The aim of this review was to analyze the published data on nonpharmacological treatments of the components of metabolic syndrome among PwS. We focused on the three most commonly recommended and used treatments in clinical practice interventions: physical activity, diet, and psychotherapy, as well as the relationship between the type of intervention and the improvement of metabolic parameters.

## Methods

This systematic review was performed in accordance with the PRISMA recommendations (16). A database search was performed in order to identify clinical trials. We included only full-text publications that met the following criteria: age of study participants ≥18 years, diagnosis of schizophrenia or schizoaffective disorder, and monitored parameters associated with metabolic syndrome. Two reviewers independently conducted literature searches using PubMed, PsycARTICLES, and the Cochrane Library databases. The keywords for the search were the following: “schizophrenia and metabolic syndrome,” “schizophrenia and physical exercise,” “schizophrenia and metabolic syndrome and exercise,” “schizophrenia and metabolic syndrome and diet,” “schizophrenia and metabolic syndrome and fitness,” and “schizophrenia and metabolic syndrome and psychotherapy.” The search was last updated on May 28th, 2018.

## Results

**Figure 1:**
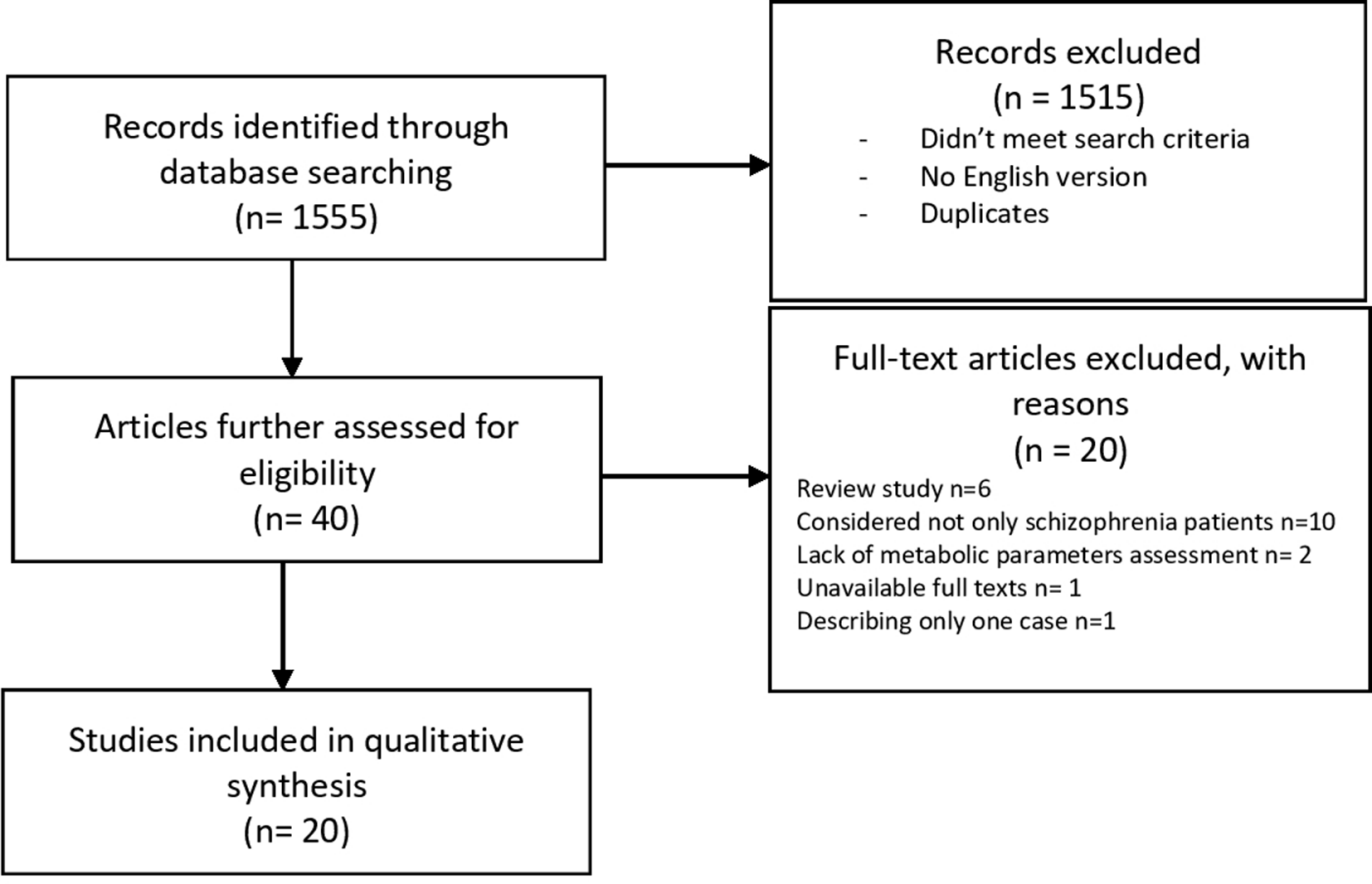
Flow diagram for the search results.

The selection process is shown in **Figure 1**. A total of 1,555 publications were found, and 40 of them met the initial criteria. Through the selection process, 20 were included in the analysis. Participants with ICD criteria for schizophrenia spectrum disorders were represented across the included studies. Studies that reported age identified the range from 18 to 65 years old. Weight (kg) or BMI (kg m^−2^) was identified as the primary outcome measure in 18 of the 20 included studies (17–34). Researchers used scales and questionnaires in order to report changes in the psychiatric domain of participants after applying interventions in nine programs (18, 23–27, 30, 32, 35). The analysis of the clinical research data presented in **Table 1** shows that authors focused on physical activity as the main nonpharmacological intervention aimed at reducing metabolic abnormalities in patients with schizophrenia. Most authors described the length, frequency, and duration of the program and the types of exercise. The intensity of training was based primarily on the values of heart rate during exercise. However, in many of the articles there was no information about the recommended exercise intensity. Participants were recruited mostly from outpatient or daily departments (17, 18, 21, 23–25, 30, 34, 36), but in almost half of the cases, the authors did not specify these data (19, 20, 26, 27, 29, 31, 32, 35, 36). Analyses of the long-term effects were conducted in four studies (21, 23, 27, 29). One study shows a correlation between the number of steps and an increase in the distance of 6-MWT immediately after the test as well as 1 month from the end (23). In a program introduced by Hsu et al. (21), the authors observed changes in the heart rate variability (HRV) parameter and body weight reduction by an average of 2.3 kg after an 8-week program. However, only weight reduction remained in the exercise group after discontinuation of the exercise program, which implies that, in patients with schizophrenia, HRV parameters are more sensitive to the effects of exercise discontinuation than body weight (21). Eight programs included a control group. All participants received APs. Study groups were highly heterogeneous in terms of age, race and ethnicity, anthropometric parameters, length of illness, and medications used. Interestingly, most of the authors did not take into account metabolic parameters in the criteria for inclusion. Only in five studies were there patients with an elevated baseline body mass index (17–21), and only one trial included the waist circumference (WC) measures (20).

**Table 1:** Short-term non-pharmacological interventions for the treatment of cardiometabolic risk factors in people with schizophrenia.

Authors (year)	Study sample (N)	Drop-out %	Study group and design of the study	Intervention	Outcome measures, assessment tools	Duration (weeks)	Effects (statistically significant)
**Methapatara et al. (က** 17 **)**	64	0	RCT Schizophrenia BMI > 23 18–65 years	Seven educational and motivational meetings. Daily walks with the pedometer. Control after 4, 8 and 12 weeks.	Body weight; BMI; WC	12	Significant differences between the experimental group and a control after the intervention: Body weight—2.21 kg (p = 0.03) BMI—0.78 kg/m^2^ (p = 0.03) Reduction of WC higher in the treatment group than in the controls (p < 0.01)
**Heggelund et al. (က** 28 **)**	25	24	Control group Schizophrenia Delusional disorder Schizoaffective disorder The same drug at the same dose 6 week prior intervention	HIT(n = 12) vs. CG (n = 7) HIT 25 min 3x/ week CG 36 min, 3x/week Supervised by a physiotherapist	VO_2peak_; e*_net_*, net mechanical efficiency of walking; BMI; RR; lipid profile; glucose; Hs-CRP	8	HIT Group: Improvement of VO_2peak_ 12% (P < 0.01) Improvement of e*_net_* 12% (P < 0.01) Improvement of HR (P < 0.01)
**Browne et al. (** 23 **)**	16	0	No control group, no randomization Schizophrenia The average age—43.3 years	Daily measurement of the activity using a pedometer. Walk in a group 2x/week. Weekly evaluation meetings. Team: coordinator, assistant Control after 4 weeks.	BMI; weight; HR; BP; 6-MWT Scales and questionnaires: PANSS; PANAS; IPAQ; MSPSS; WHOQOL-BREF; CSQ-8	10	The correlation between the number of steps and lengthening the distance in 6-MWT (immediately after the test and 1 month from the end).
**Yoon et al. (** 29 **)**	24	8,3	No control group, no randomization Schizophrenia (n = 19) Schizoaffective disorder (n = 5) Treatment with the same drug at the same dose > 4 months	Cycling 90 min/week Motivational interventions supervised by professional cyclists Control after 6 months	Weight; BMI; muscle mass; total fat; visceral fat Step test Fasting glucose Lipid profile	12	Improving cardiovascular fitness as indicated by the step test (t = 4.093, p = 0.001)
**Wu et al. (က** 24 **)**	20	20	No control group, no randomization Schizophrenia 18–55 years old	HIIT 25 min 3x/week	BMI; body weight; HR; BP; PP; MAP; PP; WC; HC; WC/HC; scales and questionnaires: PANSS; BDI; BAI	8	Body weight: 75.17 vs. 73.72 (p = 0.022) BMI: 27.76 vs. 27.21 (p = 0.022) PP: 54.11 vs. 43.33 (p = 0.01) HR: 87.33 vs. 83.83 (p = 0.01)
**Firth et al. (က** 25 **)**	32	19	No control group, no randomization First psychotic episode 18–35 years	Training at the gym —aerobic exercises —resistance exercises 45–60 min 2x/week. Team: coordinator and personal trainer.	BMI; WC; BP; 6-MWT; vertical jump Scales and questionnaires: PANSS; BDI-II; SIAS; WHODAS 2.0; WHOQOL-BREF; SOFAS; IPAQ Cambridge Neuropsychological Test Automated Battery	10	Improvement in the IPAQ score: 459 vs. 945 (p > 0.001)
**Urhan et al. (** 19 **)**	30	3	Control group (healthy) (n = 15) Schizophrenia (n = 15) Women BMI > 27 20–50 years	Personalized diet Daily physical activity (30 min walk)	BMI; body weight; WC; HC; WC/HC; glucose; lipid profile; leptin; ghrelin; insulin; HOMA-IR; body fat percentage	8	p < 0.05 Research group: Body weight: 80.88 vs. 76.85 BMI: 32.87 vs. 31.22 Body fat percentage: 40.73 vs. 38.23 Control group: Body weight: 85.43 vs. 78.64 BMI: 32.15 vs. 29.60 Body fat percentage: 41.76 vs. 37.68
**Bredin et al. (က** 26 **)**	13	31	Schizophrenia/Schizoaffective disorder 21–45 years	Moderate-to-vigorous intensity exercise 30 min, 3x/week (n = 7) Moderate-intensity resistance exercise 3x/week (n = 6)	Skinfolds; BMI; WC; lipid profile; HR; BP; maximum exercise test on an electronically braked cycle ergometer Scales and questionnaires: PANSS; CDSS; HRQOL; GLTEQ	12	2-fold increase in the duration of training GLTEQ—5-fold increase
**Kuo et al. (** 18 **)**	63	ND	Control group n = 30 Schizophrenia n = 33 BMI > 27 Non-diabetes 18–50 years	Mild/intensity exercises 20–30 min 5x/week Individual evaluation meeting Diet	WC; HC; WHR; Body weight; BMI; BP Glucose; lipid profile; HOMA-IR i HOMA-β; BDNF; adiponectin; CRP, TNF-α; IL-6; insulin, thyroid hormones; cortisol; creatinine, AST, ALT; urea OGTT BPRS	10	Body weight: 78,1 vs. 76,7 (p < 0.01) BMI: 29.5 vs. 28.7 (p < 0.01) WC: 95.3 vs. 92.7 (p < 0.05) BDNF: 4.45 vs. 9.10 (p < 0.001)
**Scheewe et al. (** 27 **)**	63	38	Control group Schizophrenia (n = 45) Schizoaffective disorder (n = 15) Schizophreniform disorder(n = 3) The average age 30 years	24 weeks Exercise vs. occupational therapy 1 h training 2x/week. Supervised by a physiotherapist	W_peak_; VO_2peak_; BMI; body fat percentage; WC; RR; lipid profile; glucose Scales and questionnaires: PANSS; MADRS	24	Increased W_peak_ of 7.6% (P < 0.01)
**Dodd et al. (က** 22 **)**	8	0	No control group, no randomization Schizophrenia	Walking 30 min/day. Aerobic exercise 30 min 2x/week Experienced coach, medical staff	Body weight; BMI; VO_2_ max; 6-MWT	24	Body weight: 75,5 vs. 73,7 (2.4%, p < 0,05) BMI: 27,2 vs. 26,6 (2.2%, p < 0,05)
**Warren et al. (က** 30 **)**	17	18%	No control group, no randomization Schizophrenia Schizoaffective disorder 18–64 years old	Walking/jogging with increasing intensity 3x/week. Educational meetings 30 min 1x/week Medical staff, coach 5 km the final running	Glucose; AST, ALT, bilirubin; lipid profile; EKG; body weight; BP; HR; Scales and questionnaires: BPRS; SANS; CGI	10	Increase in HR: 84.9 vs. 90.6 (p = 0.05)
**Sugawara et al. (က** 20 **)**	265	29%	Control group (standard care) No randomization Schizophrenia BMI ≥ 25 or WC ≥ 90 cm for males and ≥80 cm for females 20–65 years old Outpatients	3 groups: A—standard care B—doctor’s weight loss advice C—monthly individual nutritional education sessions provided by a dietitian	Body weight; BMI; WC; HR; BP; HDL-C; TG; glucose; HbA1c	48	Group C showed favorable weight loss (3.2 ± 4.5 kg)—the change differed significantly from that of group A ≥7% loss of the initial weight: 26.2% of the participants in group C 8.2% of the participants in group A Significantly lower prevalence of MetS in group C.
**Amiaz et al. (** 31 **)**	185	ND	No control group, no randomization Schizophrenia (symptomatically stable, no change in medication type and dosage for at least 6 months)	Fitness program: Group training (10 patients) with fitness instructors min 3 times/week + encouragement to exercise Diet: low calorie diet individually planned by dietitian (review once a week)	Weight; BMI; fat percentage; circumference (abdomen, thigh, pelvis, arm)	36	The mean BMI at the end of study was reduced to 28.39 ± 4.6, which was significantly lower (t = 3.352; p = 0.002) than at baseline. 13 patients showed clinically significant weight loss (reduced at least 7% of their baseline body weight) Significant reduction in fat percentage Significant reduction in: abdomen, thigh, pelvis, arm circumferences
**Hjort et al. (** 33 **)**	183		Schizophrenia Outpatient aged 18 to 45 years	Individual guidance, group sessions Part 1—physical health educational group session Part 2—1 h individual consultations Part 3—8 weeks weekly educational group session 1.5 h + practical training in the fitness room in the outpatient clinic part 4—voluntary participation in walking or running groups at the outpatient clinic part 5—education sessions for staff members *Patients were free to attend the sessions or not.	Weight, height, WC, body fat percentage (fat%), RR, HR, LDL, HDL, TG, HbA1c	120	Significant improvement in consumption of soft drinks (P = 0.001) and fast food (P = 0.009)
**Hjort et al. (** 34 **)**	54		Schizophrenia Outpatient Control group n = 6 (diet intervention)	Individual guidance, group sessions Part 1—physical health educational group session Part 2—1 h individual consultations Part 3—8 weeks weekly educational group session 1.5 h+ practical training in the fitness room in the outpatient clinic Part 4—voluntary participation in walking or running groups at the outpatient clinic Part 5—education sessions for staff members *Patients were free to attend the sessions or not.	Weight; height; WC; body fat percentage (fat%); RR; HR; total cholesterol; LDL; HDL; TG; HbA1c; VAI	120	WC 11.4 (0.7–22.1) p = 0.037 (only women)
**Kim et al. (** 35 **)**	19	0	No control group Schizophrenia Schizoaffective disorder	Group exercise moderate-intensity aerobic or resistance exercise program	TG; total cholesterol; LDL; HDL PANSS	12	No significant changes
**Hsu et al. (** 21 **)**	33	0	Training group n = 18 control group n = 15 (without exercise training) Schizophrenia Outpatients 20–60 years old BMI > 25 kg/m^2^	Week moderate intensity dance-based aerobic group exercise program conducted 2x/week 50 min Intensity (HR 60–79% of maximal) Follow up after 1 month	Body weight HRV WC HDL, TG, glucose	8	Reduced bodyweight by 2.3 kg
**Armstrong et al. (** 36 **)**	33	21%	Control group n = 17 AE group n = 16 RCT Schizophrenia and related disorders 18–55 years old	Two groups: —Treatment-as-Usual—TAU —AE individual training 1 h 3x/week Under professional trainer	RER SBP, DBP VO2 Vt PetCO2 VCO2 Wpeak 6MWT	12	Significantly increased: peak VO2, VCO2, watts, VE, and dyspnea score
**Strassnig et al. (** 32 **)**	6	0	Chronic paranoid schizophrenia (n = 4) Schizoaffective disorder (n = 2)	Treadmills 30 min 3x/week (under supervision of a research assistant)	Body weight, BMI, VO2max ml/kg/min MOS Short-Form 36	6	17% improvement in cardiovascular fitness on average (change in VO2max ml/kg/min) Significantly improved quality of life

BP, blood pressure; HR, heart rate; PP, pulse pressure; RR, the inverse of heart rate; MAP, mean arterial pressure; HRV, heart rate variability; RER, respiratory exchange ratio; SBP, systolic blood pressure; DBP, diastolic blood pressure; BMI, body mass index; WC, waist circumference; HC, hit circumference; WHR, waist/hip ratio; VAI, visceral adiposity index; 6-MWT, 6-min walking test; VO_2_ max/VO_2_peak, maximal/peak oxygen uptake; Wpeak, peak power output; VE, peak minute ventilation; Vt, peak tidal volume; PetCO2, tidal carbon dioxide pressure; VCO2, rate of carbon dioxide production at peak; OGTT, oral glucose tolerance test; HIT, high aerobic intensity training; HIIT, High Intensity Interval Training; CG, computer games; CG, control group; Hs-CRP, high-sensitivity serum C-reactive protein; HOMA-IR and HOMA-β, homeostatic model assessment-measured insulin resistance and β- cells; HbA1c, glycated hemoglobin; BDNF, brain-derived neurotrophic factor; TNF-α, tumor necrosis factor; IL-6, interleukin 6; AST, aspartate aminotransferase; ALT, alanine transaminase; RCT, Random Control Trial; e net, net mechanical efficiency of walking; HDL, high-density lipoproteins; LDL, low-density lipoprotein; TG, triglyceride; EGG, electrocardiography; PANSS, Positive and Negative Syndrome Scale; PANS, Positive and Negative Affect Schedule; BDI, Beck Depression Inventory; BAI, The Beck Anxiety Inventory; SIAS, Social Interaction Anxiety Scale; GAF, Global Assessment of Functioning; SOFAS, Social and Occupational Functioning Assessment Scale; CGI-S, The Clinical Global Impression-Severity scale; WHODAS 2.0, WHO Disability Assessment Schedule 2.0; WHOQOL-BREF, Brief Form of World Health Organization Quality of Life Questionnaire; HRQOL, Health-Related Quality of Life; IPAQ, The International Physical Activity Questionnaire; CDSS, The Calgary Depression Scale for Schizophrenia; GLTEQ, Godin Shephard Leisure Time Exercise Questionnaire; MADRS, Montgomery–Åsberg Depression Rating Scale; BPRS, The Brief Psychiatric Rating Scale; MSPSS, Multidimensional Scale of Perceived Social Support; CSQ-8, The Client Satisfaction Questionnaire; SANS, The Scale for the Assessment of Negative Symptoms; MOS Short-Form 36, The Short Form (36) Health Survey.

### Group and Individual Exercise

In eight programs, participants took part in only group exercises (21, 22, 24, 28, 30, 31, 33, 34). The analyzed studies show a reduction in BMI and body weight (21, 22, 24, 31), waist circumference (34), and an improvement in physical fitness (28).

In programs where patients were involved in only individual sessions (19, 32, 36), an improvement in physical fitness was noted (32, 36), and in one program, patients from the research group obtained a statistically significant reduction in BMI, body weight, and body fat percentage compared to the control group (19).

In five programs, combining both group exercise and individual training (17, 18, 23, 25, 29), the authors noted a reduction in metabolic or physical fitness parameters.

### Professional Supervisor

Eleven pilot programs engaged professional coaches (personal trainers/fitness instructor/physiotherapist or dietitian) who motivated patients and/or provided information and monitored the participants’ progress (18, 20–22, 25, 26, 28, 29, 31, 35, 36). In 10 of the studies, researchers obtained a statistically significant improvement in metabolic parameters or physical fitness. However, the review shows that programs without any supervisor or those that only engage medical staff without a background in fitness training/exercise can lead to similar changes in the analyzed parameters.

### Type of Exercise

Seven programs that implemented walking as a main intervention showed statistically significant changes in the tested parameters (17, 19, 22, 23, 30, 33, 34). In three of them, participants used a pedometer, which supported patients in setting individual goals and helped supervisor to track progress (17, 23, 30).

Eleven authors chose aerobic exercise to reduce metabolic syndrome risk factors and improve physical fitness in patients with schizophrenia (21, 22, 24–29, 31, 32, 35, 36). In most of them, improvement in cardiovascular fitness was achieved, and in four of them, there was also a reduction in weight and BMI. Importantly, these kinds of interventions usually require special sport facilities, such as heart rate monitors, gym equipment, and pedometers, as well as supervision by a professional trainer, which was provided in the described studies. In four programs, resistance exercise was added to basic aerobic exercises, which led to improved physical fitness but not parameters such as BMI, body fat percentage, waist circumferences, or lipid profile (25, 26, 31, 35). In nine studies, exercises were planned individually based on patient achievement, such as number of steps, maximum heart rate, or setting individual goals (17, 22–24, 29, 31, 32, 35, 36).

### Diet Intervention

Sugawara et al. applied individual nutritional education provided by a dietitian as the only nonpharmacological intervention for obese people with schizophrenia (20). Four programs applied healthy diet education as an additional intervention (19, 31, 33, 34). One study provided daily healthy meals for participants but did not specify what kind of group activity patients were involved in. This study indicated a positive correlation between blood levels of brain-derived neurotrophic factor (BDNF) and body weight reduction. Moreover, a lower level of neuropeptides was observed in people with schizophrenia compared to the control group and a statistically significant increase in BDNF after successful weight reduction (18). Results show that healthy diet educational intervention, as well as physical exercise, could reduce weight in patients with schizophrenia.

### Motivational and Educational Interventions

Thirteen authors applied a variety of motivational and educational interventions to increase compliance and hence improve outcomes (17–20, 22, 23, 25, 27, 29–31, 33, 34, 36). Six researchers conducted educational sessions where participants were introduced to healthy behavior, the importance of physical activity, and a healthy diet (17, 19, 20, 30, 33, 34). However, these interventions did not lead to better outcomes compared to those without any educational sessions. The authors encouraged patients to maintain a daily activity and diet logbook as a motivational method to maintain participation and improve metabolic parameters (18, 20, 22, 25, 27, 30, 31). Two programs offered financial incentives for participants (23, 36). In one of the studies, patients were encouraged to participate in a 10-week program, which ended with a 5-km running event (30). In two programs, one of the interventions involved increased awareness of physical health among all staff members (by education sessions for staff members). The staff’s physical health was monitored annually. Furthermore, staff members were encouraged to take part in walking and running groups on a weekly basis at the clinic. It seems that additional educational and motivational interventions did not improve results compared to programs involving only physical activity.

## Discussion

In this systematic review, a total of 20 studies implemented nonpharmacological interventions such as physical activity, dietary modification, or psychoeducation that targeted physical health aspects of metabolic and physical fitness. We found that programs that were >12 and <12 weeks can be just as effective and led to modest but significant weight loss or improved cardiovascular fitness among patients with schizophrenia. The review provides information that nonpharmacological interventions are feasible to conduct in outpatient and inpatient environments where patients with schizophrenia are hospitalized.

Nine reviews that were available as full-text articles published in English were also analyzed. Three of the nine reviews included only randomized studies (15, 37, 38). A systematic review by Bruins et al. shows that nonpharmacological interventions are more effective in the prevention of weight gain compared to weight reduction in those who already suffer from obesity. Research characterized by an individual approach to the patient was more effective than group intervention; furthermore, combined individual and group activities turned out to be the most effective in reducing obesity. Group interventions have many advantages, i.e., the imitation of behavior, mutual support, and motivation, but do not offer the opportunity to address the patient’s needs as is the case during individual sessions with a coach or therapist. Interestingly, it turned out that studies conducted in Asia were more effective than those in Central Europe, even though exercise intensity and patients’ baseline weight did not differ significantly. The authors were not able to explain these differences (15). Papanastasiou et al. analyzed 42 programs of nonpharmacological interventions targeting metabolic disturbances in schizophrenia and severe mental illness, 44 studies of pharmacological interventions, and 9 combining both methods. According to the review, a holistic approach—behavioral interventions and exercises—improved the subjective evaluation of physical health, physical fitness, and significantly increased patients’ participation in the program. The review by Papanastasiou also took into account the impact of nutritional interventions. Caloric restrictions and nutritional education seemed to be effective in weight gain prevention in several studies and mitigated weight gain in patients taking drugs such as olanzapine or clozapine. In most of the review papers, the authors failed to extract a particularly effective and distinctive intervention (39). Interestingly, Cynthia Noverand et al. suggested that there was huge potential in involving social workers due to their social skills and opportunities to work closely with patients in programs implementing nonpharmacological interventions (40). According to a meta-analysis by Chalfoun et al., in programs involving physical activity as the only intervention, aerobic interval training has a great advantage over continuous moderate exercise. Analyses of supervised programs have revealed that groups of patients with schizophrenia are able to regularly participate in such activities. However, there are some safety concerns about exercising in patients with cardiovascular disease, obesity, and low efficiency. Owing to numerous barriers and variable intensities during training, this type of physical activity in particular requires supervision by qualified personnel (41). Firth et al. in their review excluded studies involving alternative forms of activity, i.e., yoga and progressive muscle relaxation, as well as programs combining physical activity with psychosocial intervention. The average length of exercise time was 75 min per week (25–160 min/week). Of 11 experiments, 10 showed a statistically significant change in at least one of the parameters that were evaluated. Physical activity did not have a statistically significant effect on BMI; however, it significantly improved physical fitness and influenced other cardiovascular disease risk factors (42). Vancampfort et al. examined the effects of aerobic exercise on the parameters of cardiovascular performance. This study shows that an increase in VO2max/VO2peak is directly associated with clinical improvement and reduction in mortality. It has recently been agreed that poor physical fitness is a better predictor of mortality and morbidity than obesity. Body weight reduction is perceived as a big challenge in the general population. Therefore, improving parameters of physical fitness seems more realistic and easier to achieve even after a short-term intervention (43). The purpose of the review by Garcia et al. was to analyze the benefits of physiotherapy in a multidisciplinary approach in the care of people suffering from schizophrenia. The authors took into account only randomized studies and focused mainly on the impact of physical activity on psychiatric symptoms and quality of life. The results showed that aerobic exercise significantly reduced psychiatric symptoms, improved patients’ quality of life, and reduced the risk of metabolic disorders and weight gain. Interestingly, alternative forms of activity, i.e., yoga, tai-chi, and progressive muscle relaxation, significantly statistically improved patients’ mental health and quality of life. They all represent promising forms of intervention and an interesting alternative to the currently existing practices (37). In order to compare the effectiveness of various interventions, Hjorth et al. divided studies into categories (dietary interventions, *n* = 4; exercise, *n* = 5; cognitive behavioral therapy, *n* = 3; and the combination of these three interventions, *n* = 11). Reduction in body weight after the implementation of a diet was usually not maintained after the program as patients returned to their former eating habits. All tests including physical activity as the main intervention showed efficacy in term of weight reduction. Cognitive behavioral therapy also had positive effects on preventing drug-induced weight gain among patients treated with olanzapine. In all studies, the combination of three types of intervention noted a positive effect on weight reduction and some of the metabolic parameters. There was a relationship between the length of the program and its long-term effects (44). The purpose of the review by Stanton et al. was to describe the diversity of aerobic exercise and impact on the treatment of schizophrenia and schizoaffective disorder. Although the review does not include the impact on metabolic parameters and cardiovascular efficiency, it reveals the nature of the recent studies. The authors report that the variables defining aerobic exercise have not been thoroughly described. Exercise intensity was based solely on the age of the participants (38). Side effects of nonpharmacological interventions were considered to be rare and were not described in the studies.

Many studies were found to have low methodological quality, involving single-group pre–post, uncontrolled feasibility, or quasi-experimental designs. Studies predominantly used BMI as the primary outcome measure, which is a convenient and reliable measure that is predictive of health risk status (45). However, it does not reflect mass or fat distribution. Furthermore, it is likely that a decrease in fat mass and an increase in muscle mass will be less influential on BMI but more significant with regard to waist circumference. Therefore, measures of abdominal visceral adipose tissue or waist circumference should also be included as it is a measurement that indicates accumulation of abdominal fat and is associated with obesity. Very commonly, it was not clear who implemented individual interventions—the physician, therapist, nutritionist, trainer, or social worker—and who collected the data.

Caution should be taken while drawing firm conclusions from nonrandomized studies. Generally, applied interventions led to changes of metabolic and cardiovascular parameters, but most of the studies were carried out in outpatient settings where high levels of control over behavior and diet is difficult and may not be possible. Exercises were generally well described by the authors, while information on nutritional recommendations, educational and motivational interventions, or the type of implemented psychotherapy were scarce. The review suggests that adding behavioral interventions, such as educational and motivational meetings, to physical activity can bring positive results in the improvement of metabolic parameters. Walking is a form of exercise that can be performed in practically any environment, and a pedometer is a small, relatively cheap, and easy-to-use device that monitors daily activities. Patients were generally provided access to participate in exercise, but they were often encouraged to exercise on their own (17, 18, 23). Some studies suggest that medical personnel should set a good example for a healthy lifestyle, which is fundamental for “modeling” the patients (33, 34). Research shows that those medical personnel with a normal BMI are more reliable and willing to urge patients to maintain a healthy lifestyle (46).

The present studies are highly varied, especially in terms of inclusion criteria, design, type of intervention, and outcome assessment. All the analyzed studies revealed significant limitations. A common problem for nonpharmacological interventions is the high dropout rate. This was clearly illustrated in a 6-month exercise program, where participants had constant and free access to a fitness center. In this study, the percentage of dropout from the study reached 90% at 6 months and 70% at 5 months. In contrast, in the general population, this percentage is 50% at 6 months (47). One reason for withdrawing from the training program was lack of support from medical staff and families. Other important factors limiting participation in the programs are coexisting substance abuse, difficulty in making an appointment, or transfer to another health-care facility. Another reason for dropout or lack of willingness to initiate participation in the program may be the lack of an individualized approach to each patient and too many demanding exercises. The majority of recent studies suggest a need for incentives to reduce dropout from nonpharmacological intervention programs and to improve patients’ compliance. Part of the study populations was heterogeneous in term of age, race and ethnicity, anthropometric parameters, length of illness, and taken medications, which affects the representativeness of the sample. Undoubtedly, groups should be carefully selected. Most programs lacked randomization and control groups.

Future studies should certainly focus on the long-term effects that require appropriate follow-up procedures that were missing in the above-analyzed clinical trials. Engagement of a multidisciplinary team seems to be the best idea in order to optimize participation and thus achieve significant improvement and achieve long-term effects. The task of the therapists and physiotherapists would be to inform patients about alternative forms of physical activity, to provide a thorough briefing of the exercises, as well as to provide support in the process of improving individual performance and efficiency. The task of the physician and the nursing staff is to motivate patients and control for metabolic and cardiovascular parameters. Moreover, the implementation of some of the training courses is problematic due to a lack of availability of proper equipment in psychiatric departments or a lack of access to sports facilities after the end of the program. Therefore, interventions that do not require specialized sports equipment should be promoted.

## Conclusion

Patients suffering from schizophrenia can benefit from nonpharmacological interventions aimed at counteracting the CMRF. Almost all interventions appeared to have benefits for patients, either towards improving their metabolic parameters, cardiovascular fitness, or their health perception. Moreover, our systematic review provides evidence for the safety of described interventions. Future studies should comprise appropriate follow-up procedures in order to focus on the long-term effects. Study findings also show that engagement of a multidisciplinary team seems to be the best idea in order to optimize participation and thus achieve significant improvement and achieve long-term effects.

## Data Availability

All datasets generated for this study are included in the manuscript and the supplementary files.

## Author Contributions

ET and ML-S designed research; ET, ML-S, and AW were involved in data collection; ET and ML-S analyzed data; ET, ML-S, AW, and MJ participated in interpretation of findings; ET and ML-S wrote the first draft. All authors read, edited, and approved the final manuscript.

## Conflict of Interest Statement

The authors declare that the research was conducted in the absence of any commercial or financial relationships that could be construed as a potential conflict of interest.
